# Modulation of NRF2/KEAP1-Mediated Oxidative Stress for Cancer Treatment by Natural Products Using Pharmacophore-Based Screening, Molecular Docking, and Molecular Dynamics Studies

**DOI:** 10.3390/molecules28166003

**Published:** 2023-08-10

**Authors:** Abdulrahim A. Alzain, Rua M. Mukhtar, Nihal Abdelmoniem, Tagyedeen H. Shoaib, Wadah Osman, Marwa Alsulaimany, Ahmed K. B. Aljohani, Sara A. Almadani, Baiaan H. Alsaadi, Maryam M. Althubyani, Shaimaa G. A. Mohamed, Gamal A. Mohamed, Sabrin R. M. Ibrahim

**Affiliations:** 1 Department of Pharmaceutical Chemistry, Faculty of Pharmacy, University of Gezira, Wad Madani 21111, Sudannihal.khunaijir@gmail.com (N.A.); shoaibth37@hotmail.com (T.H.S.); 2 Department of Pharmacognosy, Faculty of Pharmacy, Prince Sattam bin Abdulaziz University, Alkharj 11942, Saudi Arabia; w.osman@psau.edu.sa; 3 Department of Pharmacognosy, Faculty of Pharmacy, University of Khartoum, Khartoum 11115, Sudan; 4 Department of Pharmacognosy & Pharmaceutical Chemistry, College of Pharmacy, Taibah University, Medina 42353, Saudi Arabia; msulaimany@taibahu.edu.sa (M.A.); akjohani@taibahu.edu.sa (A.K.B.A.); 5 Department of Pharmacology and Toxicology, College of Pharmacy, Taibah University, Medina 42353, Saudi Arabia; smadani@taibahu.edu.sa; 6 Department of Clinical Services, Pharmaceutical Care Services, King Salman Medical City, MOH, Al-Madinah Al-Munawwarah 11176, Saudi Arabia; bayanhs@hotmail.com (B.H.A.); ph.maryam91@gmail.com (M.M.A.); 7 Faculty of Dentistry, British University, El Sherouk City, Suez Desert Road, Cairo 11837, Egypt; shaimaag1973@gmail.com; 8 Department of Natural Products and Alternative Medicine, Faculty of Pharmacy, King Abdulaziz University, Jeddah 21589, Saudi Arabia; gahussein@kau.edu.sa; 9 Department of Chemistry, Preparatory Year Program, Batterjee Medical College, Jeddah 21442, Saudi Arabia; sabrin.ibrahim@bmc.edu.sa; 10 Department of Pharmacognosy, Faculty of Pharmacy, Assiut University, Assiut 71526, Egypt

**Keywords:** cancer, NRF2/KEAP1, oxidative stress, natural compounds, molecular docking, ADME, molecular dynamics, health and wellbeing, drug discovery

## Abstract

Oxidative stress plays a significant role in the development of cancer. Inhibiting the protein-protein interaction (PPI) between Keap1 and Nrf2 offers a promising strategy to activate the Nrf2 antioxidant pathway, which is normally suppressed by the binding of Keap1 to Nrf2. This study aimed to identify natural compounds capable of targeting the kelch domain of KEAP1 using structure-based drug design methods. A pharmacophore model was constructed based on the KEAP1-inhibitor complex, leading to the selection of 6178 compounds that matched the model. Subsequently, docking and MM/GBSA analyses were conducted, resulting in the identification of 10 compounds with superior binding energies compared to the reference compound. From these, three compounds (ZINC000002123788, ZINC000002111341, and ZINC000002125904) were chosen for further investigation. Ligand–residue interaction analysis revealed specific interactions between these compounds and key residues, indicating their stability within the binding site. ADMET analysis confirmed that the selected compounds possessed desirable drug-like properties. Furthermore, molecular dynamics simulations were performed, demonstrating the stability of the ligand–protein complexes over a 100 ns duration. These findings underscore the potential of the selected natural compounds as agents targeting KEAP1 and provide valuable insights for future experimental studies.

## 1. Introduction

Living organisms constantly experience oxidative stress, a condition considered to be one of the most significant and widespread causes of malignancies [1]. To maintain redox homeostasis at the cellular level, organisms have developed an adaptive defense mechanism against reactive oxygen species (ROS) [2]. ROS are typically by-products of cellular metabolism and oxygen consumption, generated when molecular oxygen undergoes partial reduction [3]. Within the heterogeneous group of oxygen species formed during cellular aerobic metabolism, chemically reactive radicals, such as hydroxyl (OH^−^), superoxide (O^2−^), and hydrogen peroxide (H_2_O_2_), are inevitable [4]. At low concentrations, ROS act as second messengers, playing essential roles in cell survival and various signaling activities [5]. However, excessive exposure to external sources of ROS or increased production of intrinsic ROS can lead to oxidative stress, resulting in damage to macromolecules. This oxidative stress has been associated with the development and progression of cancer [6,7,8].

The transcription factor nuclear factor erythroid 2-related factor 2 (NRF2) and its negative regulator, kelch-like ECH-associated protein 1 (KEAP1), play crucial roles in maintaining redox equilibrium [9]. Under conditions of oxidative stress, the interaction between NRF2 and KEAP1 stimulates the expression of numerous antioxidant genes. Consequently, the transient activation of the NRF2/KEAP1 signaling pathway, which protects cells from ROS, has been recognized for its beneficial effects in countering carcinogens and mutagens, and its protective role against tumor initiation in normal cells [10,11,12,13].

NRF2 belongs to the cap “n” collar subfamily of basic region leucine zipper transcription factors and is widely expressed as an antioxidant transcription factor [14,15]. KEAP1, on the other hand, is a cysteine-rich protein that regulates NRF2 function. Under basal conditions, KEAP1 mediates the ubiquitination of NRF2 through the Culins3 (Cul3)-based E3 ligase, leading to NRF2 degradation via the proteasome and maintaining low intracellular levels [16]. However, upon exposure to chemical or oxidative stress, the interaction between NRF2 and KEAP1 is disrupted, resulting in NRF2 stabilization and its accumulation in the nucleus [17,18,19]. Once inside the nucleus, NRF2 forms a dimer with small musculoaponeurotic fibrosarcoma oncogene homologue (sMAF) proteins. This complex binds to antioxidant response element (ARE) sequences, thereby promoting the transcription of genes regulated by ARE [20,21]. In addition to antioxidant genes, these ARE-driven genes include various detoxifying and metabolic enzymes, as well as regulators of inflammation [22].

The inactivation of the KEAP1-Cul3 E3 ligase pathway prevents the ubiquitination-dependent degradation of newly synthesized NRF2, leading to an increase in NRF2 levels and its translocation into the nucleus, ultimately resulting in the activation of NRF2-regulated genes [20,23]. Consequently, inhibiting KEAP1 has been proposed as a potential therapeutic approach for addressing oxidative stress-related diseases such as cancer [24,25]. Several reports have described KEAP1 inhibitors, many of which have demonstrated promising binding affinity to KEAP1 in vitro and have shown downstream effects in cellular models [24]. However, it is important to note that KEAP1/NRF2 PPI inhibitors may have some limitations in terms of selectivity [26]. Therefore, the identification and discovery of new KEAP1 inhibitors represent an effective strategy for combating the threat of cancer through targeting this pathway.

Natural products have long been utilized in the discovery and development of clinical treatments [27]. In fact, it is estimated that up to 40% of novel molecules submitted for approval by the Food and Drug Administration (FDA) are natural products, compounds derived from natural products, or compounds inspired by natural products [28]. Furthermore, epidemiological and clinical research suggests that natural products of various classes can combat oxidative stress and reduce the incidence and mortality of cancer [29,30,31,32,33,34].

Computer-aided drug design (CADD) has gained significant attention as an emerging field with the potential to accelerate and reduce the cost of drug development [35]. Among the various tools in CADD, molecular docking has emerged as an indispensable method for both CADD and structural biology, surpassing conventional approaches to drug development in terms of effectiveness [36].

Numerous compounds are being developed to target the NRF2/KEAP1 pathway, including peptide PPI modulators. These modulators help identify “hot spots” crucial for molecular binding, aiding the design of peptide or small-molecule modulators. Peptide-based targeting of PPI interfaces is commonly used as the initial approach in PPI modulator development. However, peptides are disadvantageous due to their limited ability to cross cellular membranes. Current developments have reported many small molecule inhibitors of NRF2/KEAP1. Out of these, there are naphthalene-based compounds that have been validated and exhibit good performance with potent activity [33]. More recently, a new family of indoline-based compounds was introduced as substituents for the naphthalene scaffold and it had good activity against NRF2/KEAP1 [34]. Additionally, multiple reviews comprehensively addressed the achievements and progression in the field of finding new inhibitors for NRF2/KEAP1 [35,36,37]. The current demand for direct KEAP1 inhibitors has prompted the need for a faster and more efficient drug discovery process, which can be facilitated by CADD techniques [38]. Virtual screening, in particular, holds promise in determining the binding affinity of small molecules and peptides against molecular targets, with success stories reported in various fields, including cancer research [39].

Considering the promising role of NRF2/KEAP1 signaling pathway activation in protecting cells from ROS and its potential for use in cancer treatment, this study aims to identify natural products that can modulate the NRF2/KEAP1 pathway. To achieve this, a combination of techniques including pharmacophore modeling, molecular docking, molecular mechanics/generalized Born surface area (MM/GBSA) calculations, absorption, distribution, metabolism, excretion, and toxicity (ADMET) prediction, and molecular dynamics (MD) simulations were employed.

## 2. Results and Discussion

The proper functioning of cells and tissues in the human body relies on maintaining a balance between ROS and antioxidants. When this balance is disrupted and ROS production exceeds normal levels, it leads to a condition known as oxidative stress [40]. Oxidative stress has been identified as a contributing factor, to varying degrees, in the development and progression of several diseases, including cancer [41]. In this regard, the activation of the NRF2/KEAP1 pathway emerges as a highly promising strategy to combat oxidative stress. NRF2 triggers the expression of crucial enzymes associated with the antioxidant response and various metabolic pathways [26]. From this point, our study aimed to identify small molecule inhibitors of the NRF2/KEAP1 PPI by employing multiple in silico approaches, and focusing specifically on natural inhibitors as a major target of natural compounds, due to the proven anticancer activity of phytochemicals and the recognition of the NRF2 signaling pathway [42,43].

### 2.1. Pharmacophore Modeling and Virtual Screening

**Figure 1:**
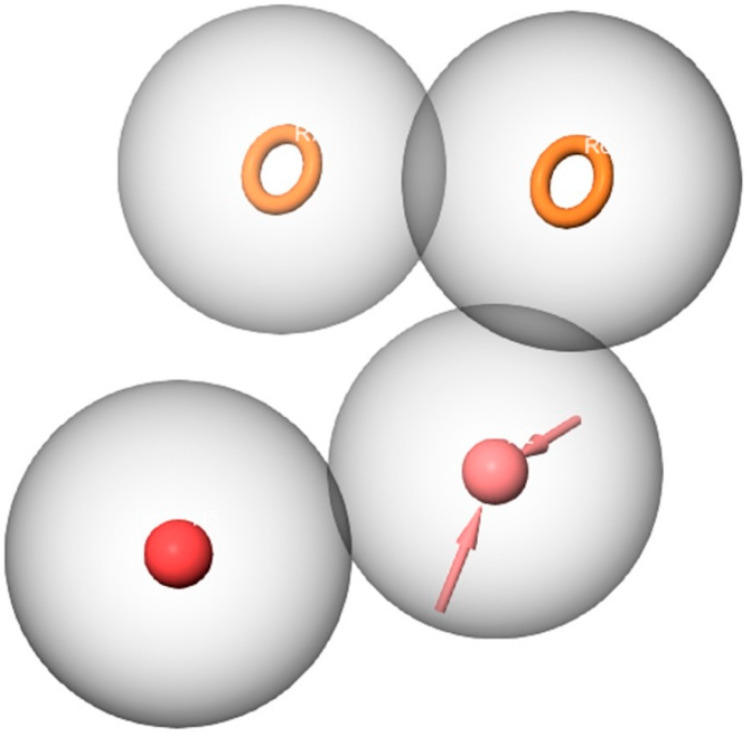
The pharmacophore hypothesis developed using the bound ligand with KEAP1 (PDB ID: 7OFE). Pink sphere with arrows, hydrogen-bond acceptor (A); yellow open circle, aromatic ring (R); Red sphere, negative ionic (N).

An energy-based pharmacophore model was generated based on the complex structure of KEAP1 with its bound inhibitor (PDB ID: 7OFE). Pharmacophore modeling has proven successful, as it reveals the essential features necessary for binding to the target site through a complementary match [44]. The generated model consisted of four essential pharmacophoric features, as depicted in Figure 1. These features included two aromatic rings (R), one negative ionic (N), and one hydrogen bond acceptor (A). To identify potential ligands, the library of 270,540 compounds from the ZINC natural products database was screened against the four pharmacophoric features. A total of 6178 compounds were identified that matched all four features and were considered for further analysis in molecular docking studies.

### 2.2. Molecular Docking and MM/GBSA Calculations

The compounds that matched the pharmacophore model (6178 compounds) underwent molecular docking using the Glide tool. Glide provides three docking methodologies, high throughput virtual screening (HTVS), standard precision (SP), and extra precision (XP). The three methodologies differ in the docking accuracy and time consumption in screening since they employ different scoring functions, with HTVS being the least accurate and fastest and XP being the most accurate and slowest. Consequently, these methodologies could be utilized sequentially to filter a large number of compounds, so that fewer compounds will be studied in the next accurate level [45]. The docking process denotes the ligand binding affinity toward the receptor in terms of docking score, which decreases as binding strength increases. In this study, the 6178 compounds were subjected to HTVS docking mode against the KEAP1 kelch domain. This resulted in 5557 compounds with docking scores ranging from 5.010 to −7.592 kcal/mol. From these compounds, the top 100 with docking scores below −6 kcal/mol, along with the co-crystalized ligand serving as a reference, were selected for further analysis using the XP docking mode.

Based on the XP docking results, 69 compounds achieved docking scores higher than the reference (<−6.633 kcal/mol). These compounds were then subjected to free-binding energy prediction using the MM/GBSA method, which considers the influence of the solvent on the binding process, providing more reliable results [46]. Overall, we identified ten compounds with better MM/GBSA dG bind energies than the reference (<−56.36 kcal/mol) as listed in Table 1. These findings suggest that these ten compounds exhibit better binding affinity than the reference compound.

The analysis revealed that ten compounds had superior docking scores and MM/GBSA dG binding energies compared to a n small molecule KEAP1 inhibitor identified by Narayanan et al. [47]. Out of the ten compounds, three (ZINC000002123788, ZINC000002111341, and ZINC000002125904) displayed the most favorable properties. These compounds achieved docking scores of −9.728, −8.259, and −7.718 kcal/mol, respectively, along with MM/GBSA dG binding energies of −72.29, −70.75, and −63.23 kcal/mol. These three compounds were selected as representatives for further analysis of interaction patterns, ADMET properties, and stability.

### 2.3. Ligand–Residue Interactions Analysis

**Figure 2:**
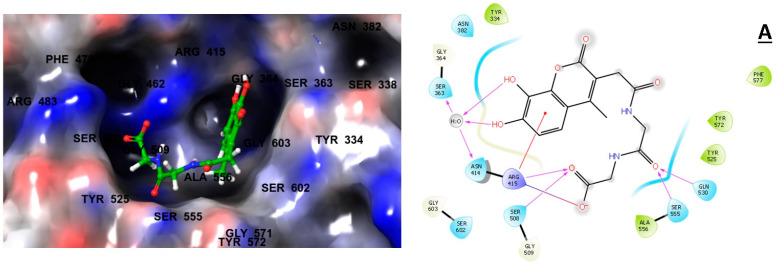

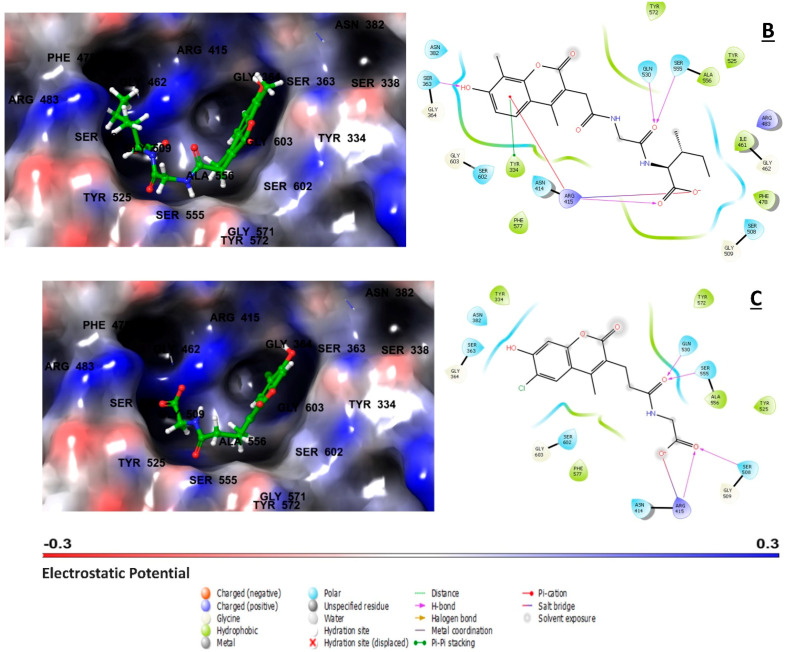
3D and 2D interactions of the top three compounds in complex with KEAP1 (PDB ID: 7OFE). (**A**) ZINC000002123788, (**B**) ZINC000002111341, and (**C**) ZINC000002125904.

We examined the interactions of the three selected compounds within the kelch domain, which comprises five sub-pockets (P1-P5). Our analysis revealed that all three compounds interacted with multiple residues from each sub-pocket, and Table 2 shows the number of interactions made with each sub-pocket. The ZINC000002123788/KEAP1 complex demonstrated a *pi*-cation interaction and a salt bridge interaction with ARG415, along with six hydrogen bond interactions involving SER363, ASN414, ARG415, SER508, GLN530, and SER555. Additionally, hydrophobic contacts were observed with TYR334, TYR525, ALA556, TYR572, and PHE577 (Figure 2A). In the ZINC000002111341/KEAP1 complex, a *pi*-cation interaction and a salt bridge interaction with ARG415 were observed, along with a *pi*-*pi* stacking interaction with TYR334. Four hydrogen bond interactions involved SER363, ARG415, GLN530, and SER555, and hydrophobic interactions were identified, including the five shared with ZINC000002123788, as well as ILE461 and PHE478 (Figure 2B). Lastly, in the ZINC000002125904/KEAP1 complex, a salt bridge interaction with ARG415 was observed, along with four hydrogen bond interactions involving ARG415, SER508, GLN530, and SER555. Similar to ZINC000002123788, hydrophobic interactions were identified (Figure 2C). Table 2 presents a comprehensive list of interactions observed between ZINC000002123788, ZINC000002111341, ZINC000002125904, and the KEAP1 kelch domain.

Previous structural studies have demonstrated that Tyr334, Ser363, Arg380, Asn382, Arg415, Arg483, Ser508, Ser535, and Ser602 are involved in binding to the Neh2 domain of NRF2 [38,48]. ZINC000002123788 and ZINC000002125904 interacted with six of these residues, three of which formed hydrogen bonds with ZINC000002123788, while only two hydrogen bonds were observed with ZINC000002125904. ZINC000002111341 interacted with seven residues, two of which involved hydrogen bonding. Moreover, the structure of Keap1 binding pocket is classified structurally as having five sub-pockets, from P1 to P5. Among them, P1 and P2 are the pockets that engrave the most polar residues (Arg483, Ser508, Ser363, Arg380 and Arg415). Meanwhile, P3 contains small polar and non-polar residues (Gly509, Ser555, Ala556, and Ser602). As for P4 and P5, they hold the hydrophobic residues including Tyr525, Gln530, Tyr572, Tyr334, and Phe577 [49]. An extensive analysis and comparison of the previously reported Keap1 inhibitors to our proposed compounds conveyed that they demonstrated similar binding modes. More specifically, our top three compounds formed hydrogen bonding and hydrophobic contacts with P1, P2, P4, and P5. Comparatively, Bertrand H. et al., reported that their top-scoring compounds displayed favorable hydrogen bonding with residues Arg380, Arg415, Arg483, and Asn382; these are present in the same pockets as our compounds [50]. In the same context, Zhuang C. et al. concluded that their findings of docking studies on noncovalent Keap1 inhibitors highlighted three important binding features: π-cation interactions, hydrogen bonds, and salt bridge interactions [51]. It is also mentioned that the different binding modes of the most active compounds, S47 and 3, with their aromatic scaffolds deeply inserted into the cationic Arg415 pocket. The aromatic portion of our studied molecules is also inserted in the pocket where Arg415 is found, l and they constituted hydrogen bonds and salt bridge with this particular residue. Therefore, we anticipate that, as long as our molecules have similar binding modes to those reported, they can present a comparable activity if studied in wet labs. Taken together, these findings provide evidence of the potential of these three compounds to inhibit NRF2/KEAP1 binding.

### 2.4. ADMET Analysis

It is crucial for new NRF2/KEAP1 inhibitors to have favorable pharmacokinetic properties, as the main challenge faced by previously identified inhibitors with high potency is their poor druggability and low bioavailability when it comes to patient use [52]. The ADMET properties of the three selected compounds were estimated using the QikProp tool in Maestro. Table 3 shows that all three compounds fall within the acceptable range for lipophilicity (QPlogPo/w), aqueous solubility (QPlogS), and cardiotoxicity (QPlogHERG) properties. However, when it comes to the CNS permeability parameter (QPlogBB), only ZINC000002123788 deviates from the acceptable range, indicating its inability to cross the blood-brain barrier. The other two compounds’ values fall within the acceptable range, suggesting their ability to cross the BBB.

In terms of gastrointestinal tract (GIT) absorption, ZINC000002123788, ZINC000002111341, and ZINC000002125904 exhibit poor absorption, as indicated by their QPPCaco values, which are less than 25 (0.206, 9.546, and 5.175, respectively). This finding aligns with the percent human oral absorption values obtained (7.256%, 52.902%, and 45.022%, respectively). However, it is worth noting that percentages between 25–80% are considered moderate rather than poor, but within the range of percent human oral absorption. These low values can be attributed to the high polarity of the three compounds. This limitation could potentially be overcome through structure optimization or the utilization of drug delivery systems. Lastly, none of the three compounds violate Lipinski’s rule of five.

### 2.5. MD Simulations

MD simulations lasting 100 ns were conducted to assess the stability of three compounds, namely, ZINC000002123788, ZINC000002111341, and ZINC000002125904, in a complex with KEAP1. The analysis included various parameters such as root mean square deviation (RMSD), root mean square fluctuation (RMSF), as well as binding interactions observed during the simulations.

**Figure 3:**
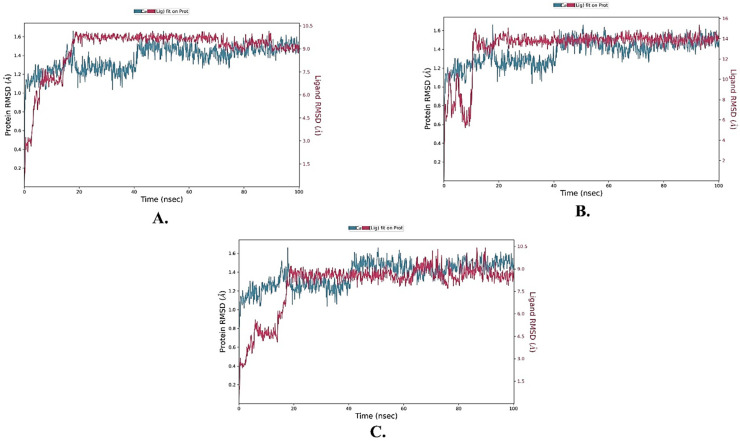
The ligand–protein RMSD plot of the top three compounds complexed with KEAP1 (PDB ID: 7OFE). (**A**) ZINC000002123788, (**B**) ZINC000002111341, and (**C**) ZINC000002125904.

Within the values of RMSD, which, in this case, measures the structural deviation from the initial conformation, a change of 1–3 Å is considered acceptable for small globular proteins. In this study, the protein’s Cα RMSD values fluctuated between 1 and 1.6 Å for most of the simulation time, with an average value of 1.372 Å (Figure 3). This indicates limited variability and stability of the protein structure. Equilibrium appeared to be reached at around 40 ns, as the fluctuations remained small within the range of 1.4 to 1.6 Å. The three ligands exhibited average RMSD values of 3.003 Å, 2.288 Å, and 1.676 Å for ZINC000002123788, ZINC000002111341, and ZINC000002125904, respectively. Their RMSD plots showed small fluctuations, with ZINC000002111341 displaying the smallest and ZINC000002125904 showing higher fluctuations.

**Figure 4:**
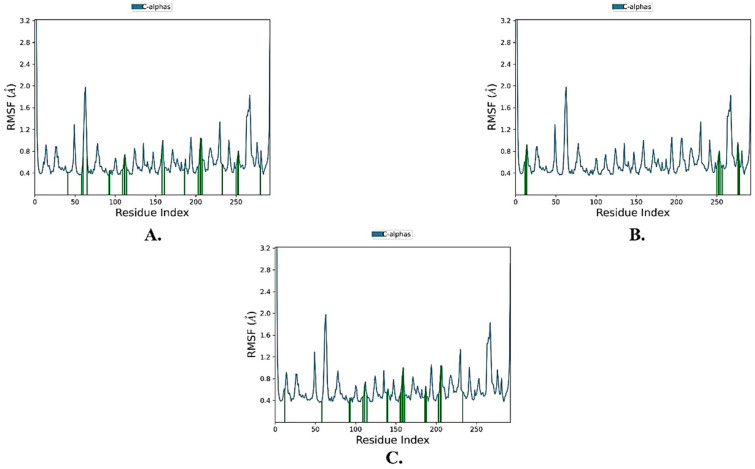
The protein RMSF plot of the top three compounds complexed with KEAP1 (PDB ID: 7OFE). (**A**) ZINC000002123788, (**B**) ZINC000002111341, and (**C**) ZINC000002125904.

Assessing the flexibility of the protein using RMSF values of Cα atoms (Figure 4), it was found that all the protein RMSF values were below 2 Å. The average RMSF values for the three complexes were 0.628 Å, indicating very limited flexibility of the protein and excellent stability.

**Figure 5:**
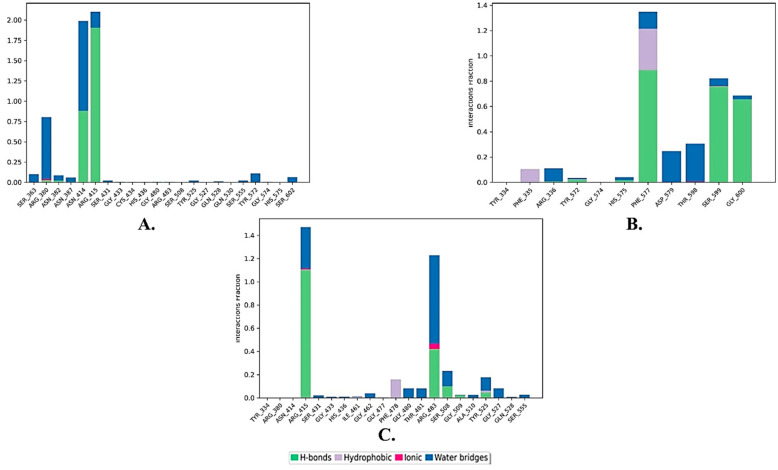
Ligand–protein contact histogram of the top three compounds complexed with KEAP1 (PDB ID: 7OFE). (**A**) ZINC000002123788, (**B**) ZINC000002111341, and (**C**) ZINC000002125904.

Regarding the binding interactions, the stability of the three complexes was primarily maintained through direct and indirect hydrogen bonds (H-bonds) (Figure 5). ZINC000002123788 formed strong direct H-bonds with ASN414 (87%) and ARG415 (190%), as well as water bridge interactions with ARG380 (70%) and ASN414 (110%). It is worth noting that some residues had interaction values exceeding 100%, which can be attributed to these residues forming multiple contacts of the same interaction type with the ligand. ZINC000002111341 exhibited strong H-bonds with PHE577 (85%), SER599 (70%), and GLY600 (65%), along with moderately hydrophobic contacts with PHE577 (40%), and water bridge contacts with ASP579 (34%) and THR598 (40%). Finally, ZINC000002125904 displayed strong and moderate direct and indirect H-bonds with ARG415 (110% and 40%) and ARG483 (40% and 80%), respectively.

Collectively, all the results support the concept that ZINC000002123788, ZINC000002111341, and ZINC000002125904 are promising candidates for KEAP1 inhibition. They outperformed the reference compound in terms of docking scores and free-binding energy, interacted with key residues, possessed acceptable ADMET profiles, and demonstrated good stability. Furthermore, the literature was reviewed in order to find any reported activities for the three compounds; according to the ZINC database, there are no reported activities found for the three compounds.

## 3. Material and Methods

The computational studies in this work were conducted using the Maestro software package version 12.8 from Schrödinger Inc. (New York, NY, USA). For the MD simulations, the Academic version of Desmond by D.E Shaw Research was employed.

### 3.1. Protein and Ligands Preparation

The crystal structure of the KEAP1 kelch domain, along with a co-crystallized small molecule inhibitor, was obtained from the Protein Data Bank with a PDB ID (7OFE). Prior to the analysis, the Protein Preparation Wizard tool was utilized for protein preparation. This involved removing solvent molecules located beyond a distance of 5 Å from the hetero groups, assigning bond orders to untemplated residues, and adding explicit hydrogen atoms to the crystal structure. The tool also employed the Prime module to add and optimize missing loops and side chains of the protein. Additionally, protonation and metal charge states for the ligands were generated at a pH of 7.0 using the Epik penalty score. Subsequently, the entire protein structure was optimized and energy-minimized using the OPLS4 (Optimized Potentials for Liquid Simulations) force field. A protein reliability report was generated to ensure the validity of the protein for further processing [37].

A library consisting of 270,540 natural compounds was obtained from the ZINC database. The MacroModel tool was utilized with default settings to process the library. To ensure the structural integrity of the compounds for subsequent computational calculations, an energy minimization process was performed using the OPLS4 force field. This step aimed to optimize the conformations and overall stability of the compounds, ensuring their reliability for subsequent computational analyses.

### 3.2. Pharmacophore Generation and Virtual Screening

Using the Phase module in Maestro, a pharmacophore hypothesis was created by analyzing the KEAP1 receptor–ligand complex [38]. This hypothesis served as a template for screening the natural product library. To ensure a stringent selection, the Ligand Screening panel within Phase was set up to exclusively identify ligands that closely matched the generated pharmacophoric hypothesis. By employing this approach, only compounds exhibiting a high degree of similarity to the hypothesized ligand–receptor interaction pattern were considered potential hits during the screening process.

### 3.3. Docking and MM/GBSA Calculations

To prepare the target for docking, a receptor grid generation step was performed to identify the ligand binding site, which is a crucial aspect of the docking process. The grid was generated using the coordinates of the co-crystallized inhibitor bound to the protein, enabling the determination of the binding site. Subsequently, the molecular docking procedure was carried out using the Glide module from the Schrödinger suite [39]. Glide offers various docking modes, such as HTVS (High Throughput Virtual Screening), SP (Standard Precision), and XP (Extra Precision), each offering different trade-offs between accuracy and efficiency. During the docking process, a scoring function was utilized to assess the strength of the binding affinity [40,41,42].

Following the docking, the Prime module, which is also integrated into the Schrödinger suite, was employed to calculate MM/GBSA values for the most promising docking poses. Prime provides researchers with a set of tools to perform MM/GBSA calculations, which estimate the free-binding energies of ligand–receptor complexes. This involved preparing input files, configuring the calculation in Maestro, executing the Prime MM/GBSA tool, and analyzing the resulting output to obtain estimates of binding free energy and identify significant energetic contributions [43,44].

### 3.4. ADMET Prediction

The QikProp module within Maestro was utilized to calculate the ADMET properties of the docked ligands. This process involved evaluating Lipinski’s rule of five and computing various descriptors to assess the drugability and safety of the designed analogues. By employing QikProp, a comprehensive analysis of the ligands’ pharmacokinetic and physicochemical properties was performed, offering valuable insights into their potential as drug candidates. Lipinski’s rule of five examines critical parameters such as molecular weight, lipophilicity, hydrogen bond donors and acceptors, and human oral absorption, among others. This rule serves as a guideline to evaluate the likelihood of a compound exhibiting favorable oral absorption and permeability [45].

### 3.5. MD Simulation

MD simulations provide valuable insights into the behavior of molecules under physiological conditions in the human body, offering a deeper understanding of the stability and mode of binding in ligand–protein complexes [46]. Furthermore, MD simulations facilitate the evaluation of non-binding interactions that occur over the course of the simulation.

In this study, the top three molecules targeting the KEAP1 protein were selected based on their docking scores, MM/GBSA energy values, and ADMET profiles. These molecules were subjected to MD simulations using Desmond v6.5 by D.E. Shaw Research. The simulation procedure began by configuring the biological system, which involved placing the ligand–protein complexes in a model of TIP3P (transferable intermolecular potential 3 points) solvent molecules within an orthorhombic box with dimensions of 10 × 10 × 10 angstroms. To achieve an electrostatically neutral state, Na+ and Cl− ions were added to the system, followed by energy minimization using the OPLS4 force field. Subsequently, the system was equilibrated using two ensembles: the isothermal-isochoric (NVT) ensemble and the isothermal-isobaric (NPT) ensemble. In the NVT ensemble, the temperature of the system was maintained at 300 K, while in the NPT ensemble, both temperature and atmospheric pressure (1 bar) were held constant. The Nose–Hoover chain thermostat was employed to maintain the desired temperature, and the Martyna–Tobias–Klein barostat methods were used to preserve the desired pressure conditions.

The production simulation was run for 100 ns, and the resulting trajectories were analyzed using Maestro’s Simulation Interaction Diagram panel [46]. This analysis provided insights into the dynamic behavior of the ligand–protein complexes and their interactions throughout the simulation time frame.

## 4. Conclusions

The NRF2/KEAP1 pathway plays a pivotal role in defending the body against oxidative stresses, making it a crucial defense mechanism. To combat oxidative stress-related diseases, such as cancer, a compelling strategy involves directly interfering with the protein–protein interaction (PPI) between Keap1 and Nrf2. In this study, we exploited a variety of computational methods for a thorough screening of numerous natural compounds to discover potential inhibitors of KEAP1. The selection process involved rigorous docking and MM/GBSA analyses, resulting in the identification of 10 compounds with exceptionally strong binding energies. Notably, three compounds (ZINC000002123788, ZINC000002111341, and ZINC000002125904) exhibited promising binding affinity to KEAP1. Moreover, we conducted ADMET analysis to evaluate the drug-like properties of these compounds, and the results indicated that they possess desirable characteristics for potential drug development. In addition, to further confirm their efficacy, we performed Molecular Dynamics (MD) simulations to assess the stability of the interactions between the identified compounds and the KEAP1 protein. Interestingly, the candidate compounds displayed stable patterns of MD profiles. These obtained results offer valuable insights into the potential of these compounds as KEAP1 inhibitors. Furthermore, their biological activity can be confirmed experimentally and investigated systematically using in vitro tests such as Nrf2 activity luciferase reporter assay, Western blotting, Reactive oxygen species (ROS) assay as well as in vivo mouse models.

## Figures and Tables

**Table 1:** Docking scores and MM/GBSA dG binding energies of the top ten compounds and the reference against KEAP1.

Compound	Docking Score (kcal/mol)	MM/GBSA dG Bind (kcal/mol)
ZINC000002123788	−9.728	−72.29
ZINC000002111341	−8.259	−70.75
ZINC000002125904	−7.718	−63.23
ZINC000096111705	−7.612	−56.53
ZINC000012530057	−7.564	−59.29
ZINC000002108268	−7.458	−60.97
ZINC000002101617	−7.130	−57.75
ZINC000000488978	−6.847	−57.77
ZINC000002126056	−6.792	−58.51
ZINC000002152231	−6.653	−60.81
7OFE-ligand	−6.633	−56.36

**Table 2:** Ligand–residue interactions of the top three compounds with KEAP1.

Compound	*Pi*-Cation	*Pi*-*Pi* Stacking	Salt Bridge	H-Bond	Hydrophobic Interactions	Other Interactions	Number of Interactions Made in Each Sub-Pocket
ZINC000002123788	ARG415	-	ARG415	SER363, ASN414, ARG415, SER508, GLN530, SER555	TYR334, TYR525, ALA556, TYR572, PHE577	Charged positive: ARG415 Polar interaction: ASN382, SER363, ASN414, SER508, GLN530, SER555, SER602	P1(2), P2(3), P3(3), P4(1), P5(3)
ZINC000002111341	ARG415	TYR334	ARG415	SER363, ARG415, GLN530, SER555	TYR334, ILE461, PHE478, TYR525, ALA556, TYR572, PHE577	Charged positive: ARG415, ARG483 Polar interaction: SER363, ASN382, ASN414, SER508, GLN530, SER555, SER602	P1(5), P2(4), P3(3), P4(2), P5(3)
ZINC000002125904	-	-	ARG415	ARG415, SER508, GLN530, SER555	TYR334, TYR525, ALA556, TYR572, PHE577	Charged positive: ARG415 Polar interaction: SER363, ASN382, ASN414, SER508, GLN530, SER555, SER602	P1(2), P2(3), P3(4), P4(2), P5(3)

**Table 3:** ADMET properties of the top three compounds.

Compound	QPlogPo/w ^a^	QPlogS ^b^	QPlogHERG ^c^	QPlogBB ^d^	QPPCaco ^e^ nm/sec	% Human Oral Absorption ^f^	Rule of Five ^g^
ZINC000002123788	−1.267	−0.752	−0.258	−3.565	0.206	7.256	0
ZINC000002111341	1.438	−2.786	−0.233	−2.099	9.546	52.902	0
ZINC000002125904	0.905	−2.373	−1.215	−1.994	5.175	45.022	0
Recommended values	−2.0–6.5	−6.5–0.5	Below −5	−3–1.2	<25 poor >500 great	<25 poor >80% high	0–4

^a^ Predicted octanol/water partition coefficient. ^b^ Predicted aqueous solubility, log S. S in mol dm^−3^ is the concentration of the solute in a saturated solution that is in equilibrium with the crystalline solid. ^c^ Predicted IC50 value for blockage of HERG K+ channels. ^d^ Predicted brain/blood partition coefficient. Note: QikProp predictions are for orally delivered drugs. ^e^ Predicted apparent Caco-2 cell permeability. Caco2 cells are a model for the gut-blood barrier. QikProp predictions are for non-active transport. ^f^ Predicted human oral absorption on a 0 to 100% scale. The prediction is based on a quantitative multiple linear regression model. ^g^ Number of violations of Lipinski’s rule of five. The rules are: mol_MW < 500, QPlogPo/w < 5, donorHB ≤ 5, accptHB ≤ 10. Compounds that satisfy these rules are considered drug-like. (The “five” refers to the limits, which are multiples of 5).

## Data Availability

The original contributions presented in the study are included in the article. Further inquiries can be directed to the corresponding author.
